# Spatially Mapping the CO_2_ Alkaline Sorbent Diffuse Microenvironment Using Operando Raman Spectroscopy

**DOI:** 10.1021/acsenergylett.5c04139

**Published:** 2026-03-11

**Authors:** Jason Pfeilsticker, Ethan Coleman, Theodore Krueger, Ankur Gupta, Wilson A. Smith

**Affiliations:** † Department of Chemical and Biological Engineering, 1877 University of Colorado Boulder, Boulder, Colorado 80302, United States; ‡ Renewable and Sustainable Energy Institute (RASEI), 1877 University of Colorado Boulder, Boulder, Colorado 80302, United States; § Department of Physics, 5237 Drew University, Madison, New Jersey 07940, United States

## Abstract

When designing a chemical process, the local balance of transport and kinetics, collectively referred to as the diffuse microenvironment, plays a critical role in performance but is difficult to directly observe. This work demonstrates a method of two-dimensional spatial chemical mapping of the diffuse microenvironment in the context of alkali metal hydroxide direct air capture of carbon dioxide using a custom operando gas-absorption flow cell along with confocal Raman spectroscopy. Notably, we observe the concentration boundary layer near the gas–liquid interface and elucidate the interplay of carbonate and bicarbonate ions within it while inferring local hydroxide depletion through continuum modeling. These first of their kind observations provide a technique to compare the performance of direct air capture solvents based on diffuse microenvironment dynamics while also providing metrics important for air contactor design such as boundary layer thickness. Overall, this work showcases a new experimental platform to study interfacial diffuse microenvironments in and outside of the field of direct air capture of carbon dioxide.

## Graphical abstract

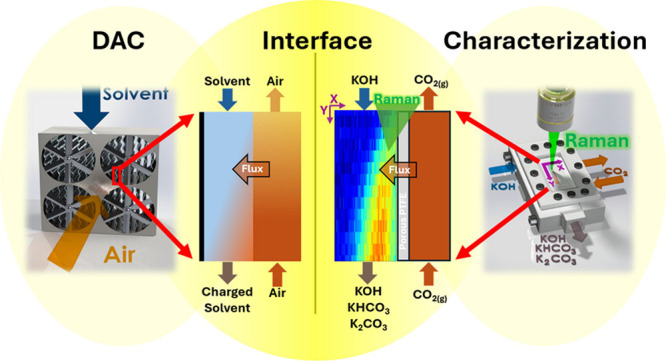

A large number of chemical processes and industrial unit operations are facilitated by the formation of, or transport through, boundary layers. Notable examples are membrane based separations and gas absorption. Absorption of carbon dioxide (CO_2_) into a liquid provides an excellent case study of a concentration boundary layer. The modern economic drive for large scale computing and its insatiable demand for reliable and rapidly dispatchable electricity is increasingly being met with on-site natural gas turbine generation.^1^ Increasing atmospheric CO_2_ concentrations are hypothesized to bring on social, infrastructure, and economic hardship potentially leading to global instability.^2,3^ These projected concerns provide generational existential motivation to reduce atmospheric CO_2_ levels which are currently at a concentration of 428 ppm.^4^ As of 2022 it is estimated that there are 3240 metric gigatons of CO_2_ in the atmosphere making it a relatively abundant carbon source.^4–7^ Therefore, the direct air capture (DAC) of atmospheric CO_2_ provides an opportunity to create a circular economy and supply chain of the future by valorizing the mitigation of a potential hazard into market opportunities. The field of DAC has been growing steadily over the past decade, moving out of the lab into real world deployment across the world. As of this publication, Climeworks’ Mammoth facility is the largest operating DAC plant in the world and utilizes a solid amine adsorbent to facilitate a designed capture rate of 36,000 tons of CO_2_ per year.^8^ This is soon to be eclipsed by 1PointFive’s Stratos facility currently under construction in Texas, which is designed to remove 500,000 tons of CO_2_ per year via aqueous alkali-metal hydroxide carbonate cycling.^9^ Alkali-metal hydroxide based CO_2_ absorption is directly tied to early characterization of CO_2_ by Joseph Black in 1755 and has been industrially studied since the early 20th century, however the first notable mention of this approach in a climate change context was stated by Klaus Lackner in 1999.^10–12^ The main drawback of these types of systems is the large energy penalty to recover pure CO_2_ from carbonate species while also regenerating the alkaline absorbing solution to create a closed loop cycle.^13,14^ In an effort to reduce the recovery energy penalty, the concept of reactive capture and conversion (RCC) was developed. In RCC schemes, a liquid solvent, typically an amine, hydroxide, or ionic liquid, is put into contact with air from which CO_2_ is absorbed. This CO_2_ rich solvent stream is then directly utilized in chemical reactions to form CO, CH_4,_ or other higher value products via thermal or electrocatalytic processes, combining the solvent regeneration, CO_2_ release, and the CO_2_ conversion steps to ideally minimize the capital and operating costs of a DAC plant.^15^ RCC schemes typically utilize liquid DAC solvents as opposed to solid DAC sorbents to facilitate continuous operation. Electrocatalysis is of particular interest for RCC due to the potential for precatalytic activation and microenvironment engineering by the RCC solvent, and the ability to integrate with renewable energy.^16^ With many DAC and RCC solvents competing for practical viability, measurements that allow direct spatial and chemical characterization of the absorption process at the gas–liquid interface would be useful to compare solvents under relevant, flowing, DAC conditions to accelerate their development and deployment. Previous works have explored mapping microenvironments and their effects on electrochemical CO_2_ reduction, but have not studied the two-dimensional down-channel perspective or in the context of DAC.^17,18^ This work demonstrates a novel methodology using a custom-designed 3D-printed operando air-contactor flow-reactor and confocal Raman-spectroscopic spatial-mapping to collect two-dimensional steady state concentration profiles of contactor boundary layers of an alkaline air capture solvent in contact with CO_2_. The first of their kind 2D maps experimentally reveal the interplay between transport and equilibrium chemistry in a flowing DAC processes providing a platform for DAC solvent performance characterization and speciation determination in multistep equilibrium capture processes. We also demonstrate qualitative similarities of the experimental measured profiles with a custom Python-coded 2D model, which serves as a starting point for further development that can help elucidate deeper kinetic and transport insights. Overall, this work presents the first 2D spatial characterization of the diffuse interfacial microenvironment in direct air capture solvents under relevant operating conditions and provides a unique and powerful platform for assessing and co-optimizing novel DAC solvents and air contactor systems with potential applications in a variety of diffuse microenvironment transport processes.

A bench-scale membrane-based air contactor with a gas–liquid interfacial area of 48 mm^2^ was developed to facilitate 2D hyperspectral mapping of mass transport boundary layers of CO_2_ contacting potassium hydroxide (KOH) of different concentrations and flow rates via a porous PTFE membrane. Detail discussion regarding cell design choices can be found in the Supporting Information. A rendering of the flow cell undergoing confocal Raman spectroscopic mapping and a top-down schematic depiction of the mapped XY plane at the interface are shown in Figure 1d,c analogously compared to a large scale contactor and its interface in Figure 1a,b.

**Figure 1:**
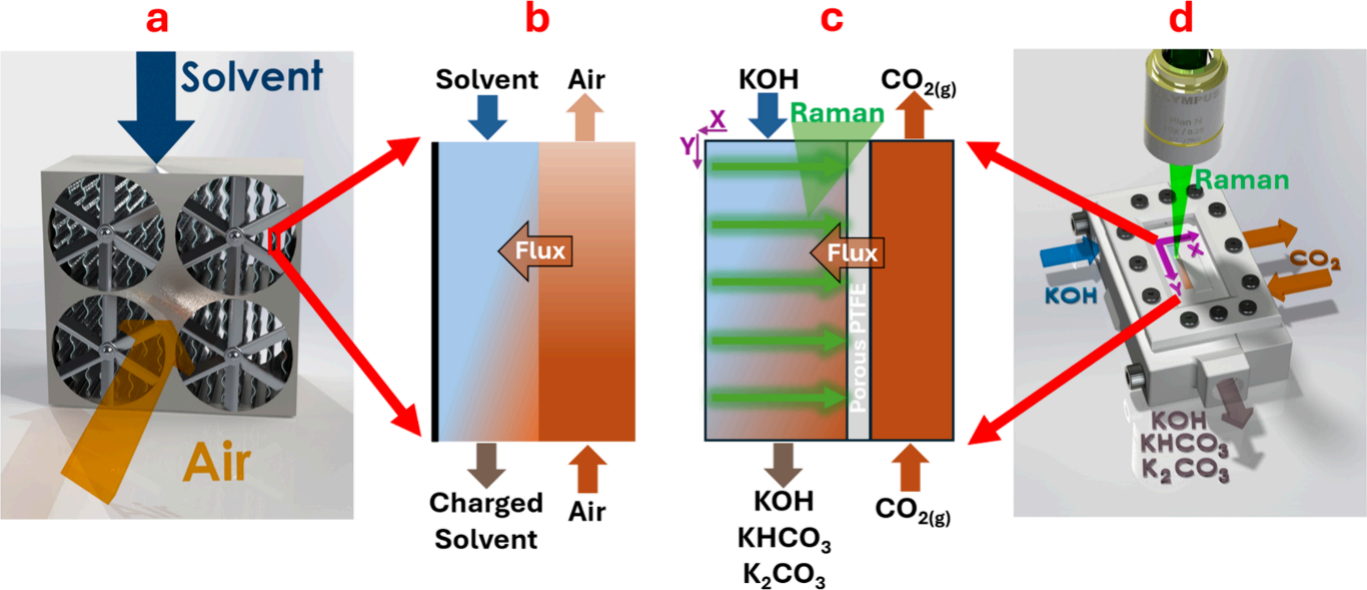
(a) Rendering of industrial DAC air contactor. (b) Cartoon depicting zoomed in CO_2_ absorption interface for a real-world DAC contactor. (c) Cartoon depicting zoomed in CO_2_ absorption interface in this work’s operando reactor with the green arrows depicting the raster pattern along which Raman spectra are measured. (d) Rendering of this work’s operando reactor undergoing Raman mapping.

Pure CO_2_ gas was flowed through the gas side of the flow cell using a mass flow controller (Alicat MC-500SCCM-D/5M) and KOH solutions were flowed through the liquid side of the flow cell using a syringe pump (Chemyx fusion 200). When KOH contacts CO_2_ through the PTFE membrane, we assume that the CO_2_ is consumed through a simplified cascade of equilibrium reactions, eqs 1–5. shown below:

1 $$H_{2}O\overset{k_{1}^{f}}{\underset{k_{1}^{b}}{⇌}}{\mathrm{OH}}^{-}+H^{+}$$

2 $${\mathrm{CO}}_{2\left(\mathrm{aq}\right)}+{\mathrm{OH}}^{-}\overset{k_{2}^{f}}{\underset{k_{2}^{b}}{⇌}}{\mathrm{HCO}}_{3}^{-}$$

3 $${\mathrm{CO}}_{2\left(\mathrm{aq}\right)}+H_{2}O\overset{k_{3}^{f}}{\underset{k^{b}}{⇌}}{\mathrm{HCO}}_{3}^{-}+H^{+}$$

4 $${\mathrm{HCO}}_{3}^{-}+{\mathrm{OH}}^{-}\overset{k_{4}^{f}}{\underset{k_{4}^{b}}{⇌}}{\mathrm{CO}}_{3}^{2-}+H_{2}O$$

$$H^{+}+{\mathrm{CO}}_{3}^{2-}\overset{k_{5}^{f}}{\underset{k_{5}^{b}}{⇌}}{\mathrm{HCO}}_{3}^{-}$$ 5

Carbonic acid has been eliminated from the reaction scheme due to its extremely short lifetime and lack of spectral evidence for its presence. The flow cell was supplied with fresh KOH from a reservoir which was continually sparged and blanketed with Argon gas to prevent adventitious CO_2_ absorption. Spectroscopic measurements were performed using a Horiba XploRA PLUS confocal Raman microscope equipped with a precision automated XY stage using a 94.7 mW 532 nm excitation laser with an Olympus MPlan 10X 0.25 NA objective yielding an approximate voxel size of 3 μm diameter and 34 μm height. To construct concentration maps from raw hyperspectral images, calibration curves were generated via in situ spectroscopic titrations of 0.1 M bicarbonate with argon sparged/blanketed 1 M KOH. Calibration curves were calculated for the 1014 cm^–1^ and 1064 cm^–1^ C–O stretches for bicarbonate and carbonate by fitting Voigt lineshapes.^19^ An example of calibration curve generation can be seen in Figure SI2 with more detailed commentary regarding ionic strength and activity found in the Supporting Information. For mapping, spectra were captured using a precision XY stage in a raster pattern. Each XY spectra was processed through respective calibration curves to construct concentration maps as demonstrated below in Figure 2 with an example of a 2D carbonate map shown in Figure 2d.

**Figure 2:**
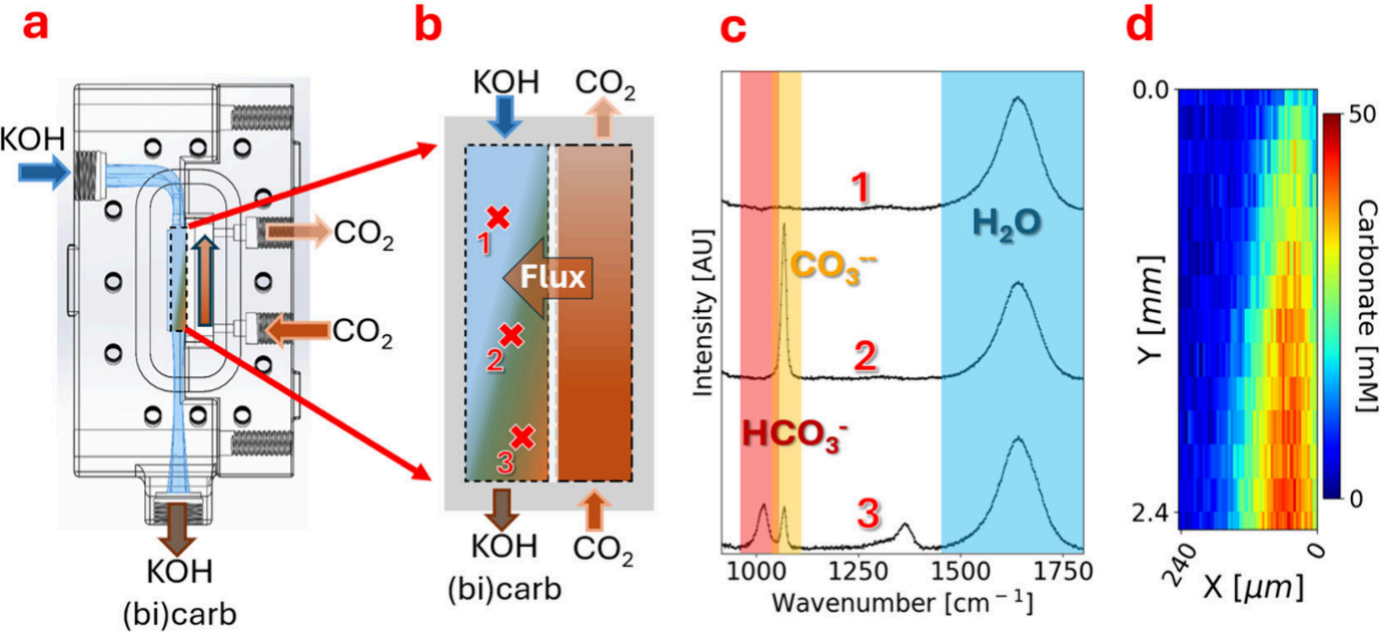
(a) Top-down operando reactor image. (b) CAD rendering showing flow paths. (c) Zoom in on interface highlighting three Raman measurement points (1, 2, 3). (d) Raman spectra associated with points 1, 2, and 3 with bicarbonate (red), carbonate (orange), and water (blue) peak regions labeled and highlighted. (e) Example carbonate concentration map as calculated from the carbonate peak at many XY positions.

To aid in understanding the experimental data, a 2D continuum model was developed assuming the reactions in eqs 1–5. The assumptions and boundary conditions are discussed further in the Supporting Information consummating in a system of five coupled partial differential equations the general form of which is shown in eq 6. The fully expanded forms are shown in the Supporting Information eqs 8–12:

6 $$V_{y}\left(x\right)\frac{\partial C_{i}}{\partial y}=D_{i}\left(\frac{\partial^{2}C_{i}}{\partial y^{2}}+\frac{\partial^{2}C_{i}}{\partial x^{2}}\right)+\underset{j}{\sum }\left(k_{j}\underset{n}{\prod }{\left(\gamma_{j,n}C_{j,n}\right)}^{v_{j,n}}\right)$$

Where *V*_*y*_ (*x*) is the Y direction velocity in m/sec as a function of X position, *C*_*i*_ is the *i* ^*th*^ species concentration in mol/m^3^, *D*_*i*_ are the *i* ^*th*^ species diffusion coefficient in m^2^/sec, *k*_*j*_ is the rate constant for the *j* ^*th*^ reaction, *γ*_*j,n*_ is the *n* ^*th*^ species activity coefficient calculated from the extended Debye–Hückel equation involved in the *j* ^*th*^ reaction, is the *n* ^*th*^ species involved in the *j* ^*th*^ reaction, and *ν*_*j,n*_ is the *n* ^*th*^ species reaction order in the *j* ^*th*^ reaction. Notably missing are electromigration terms. This will be discussed along with our experimental results below.

Spectroscopic spatial mappings were performed under liquid flow rates of 0.5, 1.0, and 1.5 mL/min and KOH concentrations of 0.1 M and 0.2 M. These flow rates correspond to maximum in channel velocities of 1.1, 2.5, and 3.8 mm/sec which are in good agreement with Keith et. al’s seminal work.^13^ Due to the method used to hold the membrane they tended to bow out into the liquid channel as depicted in Figure 3a,c. To counter act this, design measures were taken, but we could not fix the bowing without compromising the homogeneous porosity of the membrane. A post processing method was developed to remove the bowing and create rectilinear maps. The process row-wise locates the x-position where the CF_2_ stretch at 731 cm^–1^ from the membrane onsets and horizontally shifts each row to align at that PTFE onset X location. An example of unshifted and shifted maps are shown below in Figure 3c,d. A mapping showing the PTFE band can be found in supplementary Figure SI4. The typical maximum horizontal shift was on the order of 400 μm.

**Figure 3:**
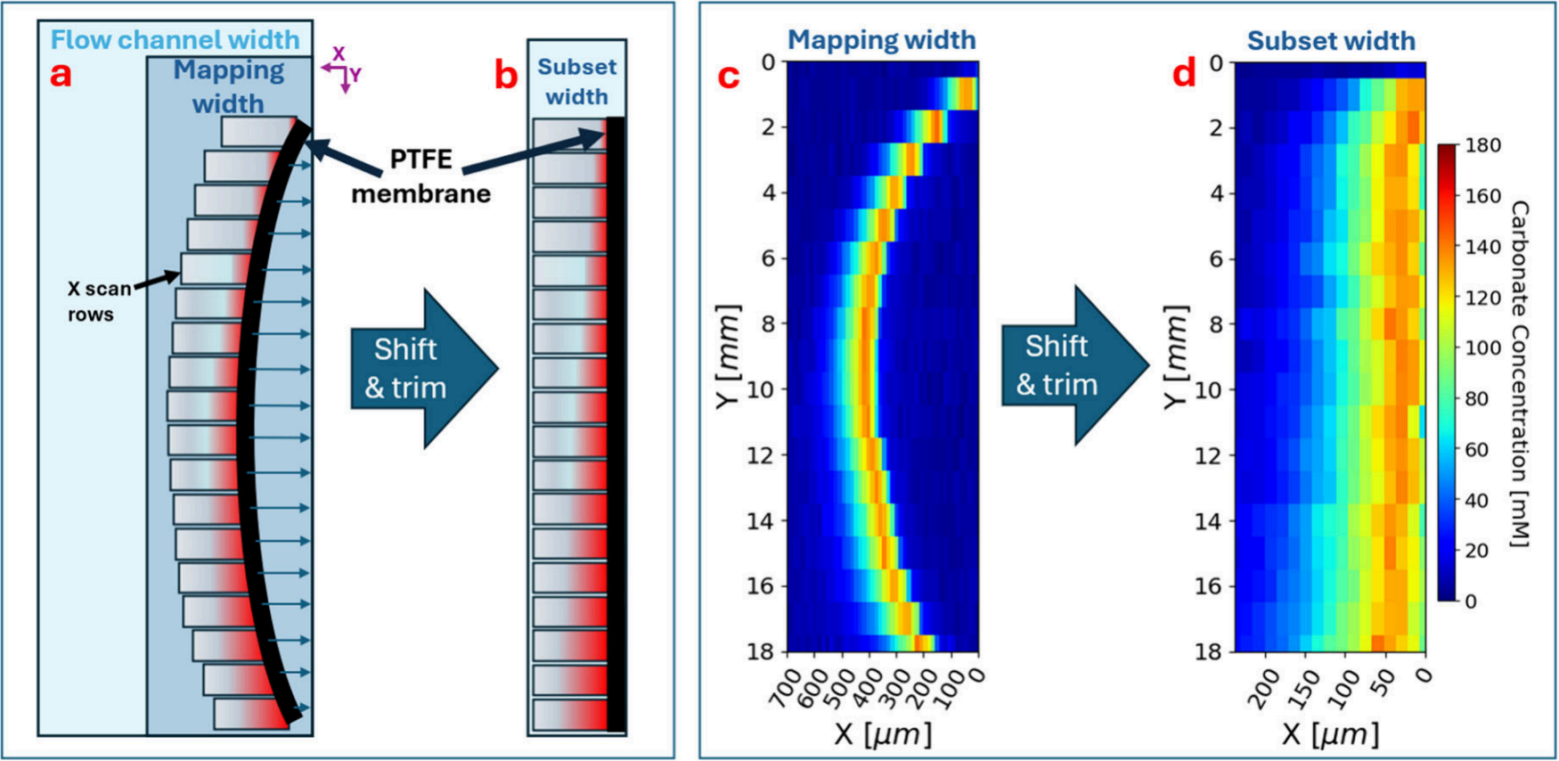
(a) Cartoon depiction of liquid flow channel of the flow cell with the membrane bowing out and spectroscopy X scan rows. (b) Cartoon depiction of shifted and trimmed map X scan rows. (c) Example of raw carbonate map and (d) its shifted and trimmed version. Note that the whole channel is not mapped and that the shifted X scan rows are equally trimmed from the left to the width of the smallest subset where the maximum membrane bowing occurs. Also note the aspect ratios of the maps are not 1:1 and thus accentuate the membrane bowing.

By varying the concentration and flow rate of the alkaline DAC sorbent, we were able to observe various microenvironments of the direct air capture process. In all cases, as seen in Figure 4 at the top right corner where fresh KOH first contacts CO_2_, there is a very sharp increase in carbonate concentration as there is an excess of hydroxide ions to rapidly push through reactions shown in eq ’s 2 and 4 to the terminal carbonate species. This locally high concentration of carbonate causes a diffusive flux, resulting in the X direction gradient in carbonate concentration moving away from the membrane. This initial exposure locally depletes the hydroxide concentration near the membrane. As a consequence of this hydroxide depletion and as the concentration profile develops down the flow channel, solubilized CO_2_ must diffuse further away from the membrane to react with hydroxides which are slowly replenished through diffusive fluxes from further into the bulk toward the left side of the liquid flow channel. Interestingly, the equilibrium between carbonate and bicarbonate shifts toward bicarbonate near the membrane where hydroxide is locally depleted which relaxes the forward push through the equilibrium cascade. That is to say the transport facilitates a local pH gradient from high to low moving away from the membrane. This apparent reversal of the equilibrium cascade is exacerbated down the reactor due to increased local hydroxide depletion near the membrane. The observed result of this is that the X location of the maximum carbonate concentration moves further away from the membrane moving down the channel and a thin layer of maximum bicarbonate concentration nests itself between the carbonate-maximum and membrane.

**Figure 4:**
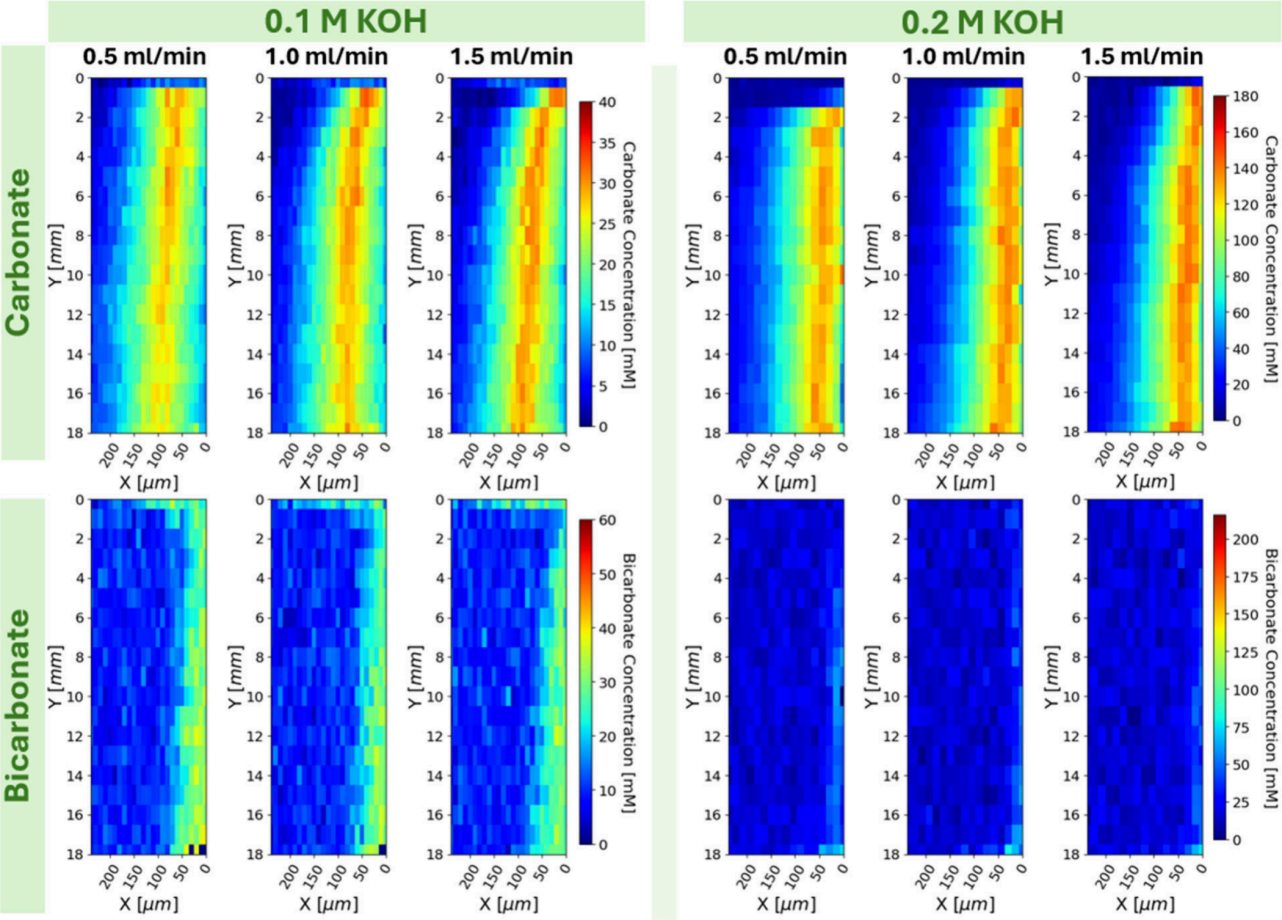
Carbonate and bicarbonate maps (top and bottom rows) at 0.1 and 0.2 M KOH (left and right blocks) at flow rates of 0.5, 1.0, and 1.5 mL/min (columns). The horizontal bands in the 0.1 M bicarbonate maps are due to polymer fluorescence near the edge of the back reflector. Liquid flow is top to bottom with the membrane at the right edge of the map.

This somewhat counterintuitive experimental result demonstrates that our method is able to resolve the interplay of equilibrium chemistry, convective fluxes, and diffusive fluxes within a diffuse microenvironment near an interface within context of a DAC contactor.

Changing the flow rate of the KOH from 0.5 mL/min to 1.5 mL/min had effects on the development length for the boundary layer for both 0.1 and 0.2 M KOH, though the full channel-length maps in Figure 4 are not high resolution enough to capture that development difference within the first 2500 μm. The high resolution maps in Figure 5 focus on the start of the boundary layer formation within the first 2500 μm of the liquid channel where significant differences are seen. The curvature at the beginning of the boundary layer is tightest in the 0.5 mL/min map and longest in the 1.5 mL/min map as expected given the velocity profile and thus momentum boundary layer differences.

**Figure 5:**
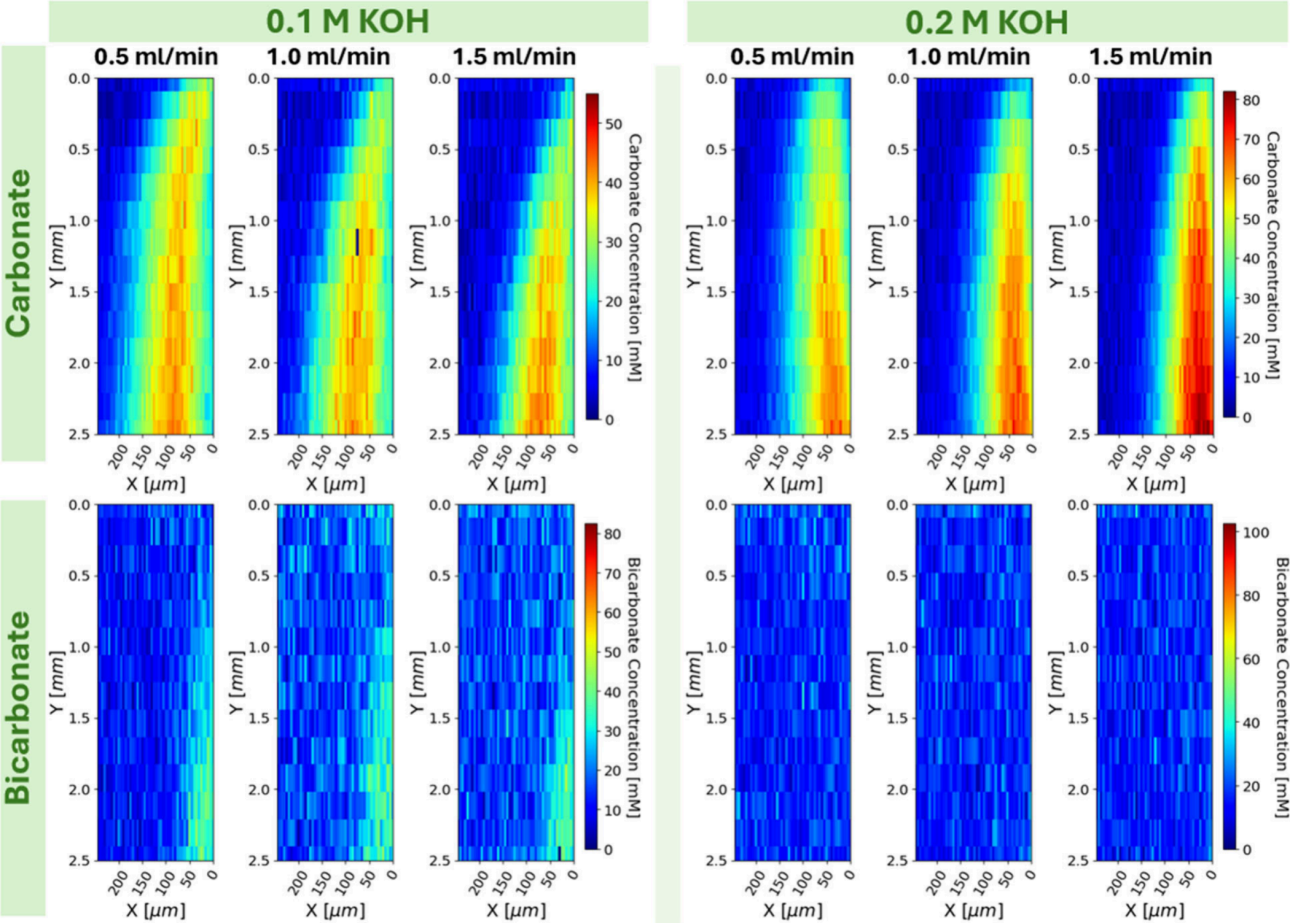
High resolution maps of the first 2.5 mm of the clow channel of Carbonate and bicarbonate (top and bottom rows) at 0.1 and 0.2 M KOH (left and right blocks) at flow rates of 0.5, 1.0, and 1.5 mL/min (columns). Liquid flow is top to bottom with the membrane at the right edge of the map.

At the lowest flow rate, only slightly higher bicarbonate concentrations were seen near the membrane compared to the highest flow rate as seen in Figure 5. This can be explained by higher effective local residence times and by greater local hydroxide depletion near the membrane. Doubling the KOH concentration from 0.1 to 0.2 M increased the maximum carbonaceous concentration and skewed the equilibrium toward carbonate. Notably, the increased hydroxide concentration seems to have alleviated hydroxide depletion near the membrane. As a result of high OH^–^ flux, the thin interstitial bicarbonate layer is significantly diminished and closer to the membrane.

To understand the dynamics of these operando experimental measurements, the previously discussed computational model was deployed. The model was run for each condition with literature or thermodynamically consistent values for all parameters and our measured velocity profiles which can all be found in supplementary Table SI1 and Figure SI1 respectively. Side by side map/model comparisons are shown below in Figure 6. The computational model also produces the thin layer of bicarbonate due to hydroxide depletion near the membrane demonstrating good experimental agreement. Hydroxide maps can be found in Figure SI5. Quite noticeably though, the initial contact with the membrane at the top right of the model maps shows a carbonate concentration hot spot which is missing in the experimental maps. The model also predicts higher concentrations over the entire domain than experimentally observed. We suspect this is because our model does not account for electromigration, which we suspect plays a significant role. This is highlighted when the molar charge concentration is plotted as in Figure SI5. Most notably, where the carbonate concentration hot-spot is observed, the charge concentration also has a corresponding nonphysical hot-spot. If electric fields were simultaneously solved for while including electromigration terms in the governing equations, these issues would likely be rectified. Attempts were made and are ongoing to program a full electroneutral-Nernst-Plank python model, but for the purpose of understanding the carbonate-bicarbonate shifts and local hydroxide depletion near the membrane, this facile model seems satisfactory.

**Figure 6:**
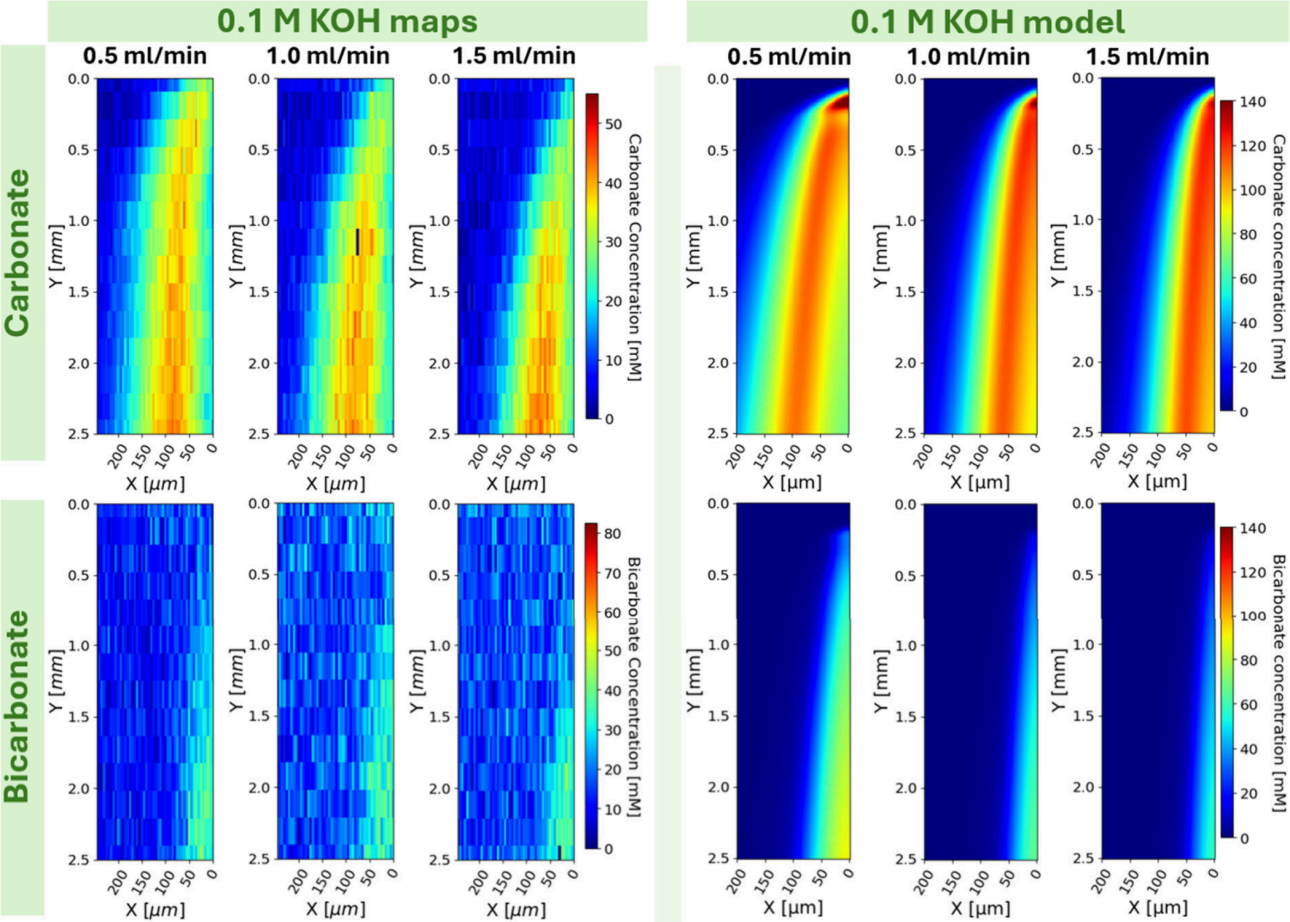
Left block: Experimental bicarbonate and carbonate maps of 0.1 M KOH flowing at 0.5, 1.0, and 1.5 mL/min. Right block: Modeled bicarbonate and carbonate maps at the same conditions as the experimental maps and trimmed to the same spatial domain as the experimental maps. Liquid flow is top to bottom with the membrane at the right edge of the map.

With a higher fidelity model, it is possible that the inverse problem via model fitting to the experimental data could be utilized to extract the kinetic and transport parameters to compare DAC solvents under real world flowing conditions enabling the acceleration of their development and deployment. The large parameter space and range of kinetic constants make the inverse problem a nontrivial task. Solving the model many times to perform fitting is computationally expensive and beyond the scope of this initial work, though perhaps this is an opportunity to apply physically informed neural networks or other machine learning tools.^20,21^

Given that the concentration maps are largely a function of the DAC solvent, we propose that our mapping method can be used as a screening method and tool to better understand novel DAC and RCC solvents with potentially more complicated equilibria. The absorption chemistry is only part of the picture for contactor design. Our method and the first of their kind 2D concentration maps can provide the full transport picture allowing for the optimization of film thickness in contactors at scale through the trade-off of contactor area versus flow rate via the arguments of local hydroxide, or general solvent, depletion discussed above. These maps can also help build structure property relationships to help steer DAC and RCC solvent synthesis efforts. Given that this technique can resolve diffuse microenvironments, one could imagine a plethora of applications. We leave the reader with a few examples: understanding heterogeneous electrocatalytic diffuse microenvironments, device flow architecture, and their role in selectivity, performance, and optimization of operating conditions; mapping local pH for interfacial processes via ratios of buffer species, understanding homogeneous electrocatalysis speciation and spatial distribution of electron transfer processes while enabling closing of mass balances due deactivation; understanding the chemical effects and implications of corrosion processes related or unrelated to electrocatalysis; and understanding local transport in interfacial processes such as those in modern and novel approaches critical mineral separations. We expect our mapping method to advance the understanding of diffuse microenvironments and their impact and metrologic importance in DAC and many other fields.

## Supplementary Material



## Notes

Detailed discussion on cell design, modeling, spectroscopic titrations, apparent p*K*_a_, and activity; figures supporting claims and observations; and table of modeling constants (PDF)
