# Supplementary material for: Spatially Mapping the CO2 Alkaline Sorbent Diffuse Microenvironment Using Operando Raman Spectroscopy
Source: ACS Energy Lett. 2026 Mar 11;11(4):3258–64. doi: 10.1021/acsenergylett.5c04139 (PMC13077696; doi:10.1021/acsenergylett.5c04139)
Supplement: Supplementary file 1 [file nz5c04139_si_001.pdf]

# Spatially mapping the $CO_2$ alkaline sorbent diffuse microenvironment using operando Raman spectroscopy

Jason Pfeilsticker[1,2], Ethan Coleman[1], Theodore Krueger[3], Ankur Gupta[1] and *Wilson A. Smith[1,2]

[1] Department of Chemical and Biological Engineering, University of Colorado Boulder, Boulder, CO 80302

[2] Renewable and Sustainable Energy Institute (RASEI), University of Colorado Boulder, Boulder, CO 80302

[3] Department of Physics, Drew University, Madison, NJ 07940

*Corresponding author: Wilson.Smith@colorado.edu

**Methods detailed discussion:**

The majority of the flow cell was resin 3D-printed using Syria Tech Sculpt clear resin which can tolerate cleaning via boiling in water and spectroscopically appears to be chemically stable under this work's operating conditions. While a real-world scalable DAC air contactor would typically have an unmediated interface between the air and the sorbent as depicted in Figure 1a, our reactor utilizes a porous PTFE membrane (CS Hyde EAP040) to maintain horizontal bulk phase separation to facilitate top-down spectral mapping. Having a membrane allowed us to mechanically stabilize the gas liquid interface such that we could take spectra without the inface moving. While the gas-liquid interface in a real-world contactor and ours may differ slightly, the underlying process is the same as highlighted in Figure 1b,c. The membrane splits the reactor into two flow channels, where the left channel (as depicted in all Figures) is for the liquid DAC solvent and the right channel is for the gaseous $CO_2$ stream. The liquid side of the flow cell is the only externally visible portion of the cell and is where the chemical mapping is performed.

The side-by-side orientation of the spectral characterization flow cell was chosen due to a tradeoff between the geometric and optical constraints of the numerical aperture of the microscope objectives and their working distances as well as our instrument's preferred rastering method. We desired to have a long optical working distance, which typically results in a tall narrow voxel, thus mapping in the XY plane was preferred to maximize how close to the edge of the flow cell the measurements could get before losing signal by partial eclipsing of the light cone by the measurement chamber's wall. The top of the flow cell was sealed with a nitrile O-ring and a clear 1.5 mm thick borosilicate glass window through which Raman mapping takes place with minimal optical losses. Care was taken while designing the flow cell and the associated hardware to minimize the possibility of bubble formation and accumulation while maximizing flow path smoothness such that the cell could be reliably operated at steady state, under fully developed laminar flow conditions, and without optical interruption from bubbles. To ensure laminar flow operation, particle tracking velocimetry was performed using dilute suspended 3 micron diamond particles (Allied 90-3DL3). Automated particle tracking was performed via the TrackMate ImageJ plugin, though inconsistent results were found due to

algorithmic particle detection errors and frame rate aliasing. Manual frame-by-frame particle tracking was thus performed from which velocity profiles were calculated as shown in Figure SI1. The quantitative quadradic fit indicates laminar Hagen-Poiseuille flow of the KOH solution within the XY plane. Confirming laminar flow and having these velocity profiles will be required to make meaningful analysis of $CO_2$-sorbent interactions and to develop accurate transport models and will be discussed later in the modeling section. A lower signal to noise ratio was observed when flowing the KOH at 1.0 and 1.5 ml/min and is attributed to the 60 Hz frame rate of the camera (IDS UI388xLE-C) limiting the number of frames the velocity could be averaged over given field of view limitations.

To ensure adventitious CO2 would not enter the reservoir KOH during mappings and titrations it was excessively blanketed with argon. To check this was the case, control Raman spectra were taken of the reservoir KOH before and after mappings to check for the absence of bicarbonate and carbonate.

A key feature of the flow cell is the incorporation of a thin 316 stainless steel back-reflector into the bottom of the measurement chamber to remove polymer fluorescence signal from the bottom of the liquid flow channel.

When applying baselines to spectra, the 1640 $cm^{-1}$ water bending mode peak was used as an internal standard to ensure applications were consistent for all spectra as its shape and intensity are minimally affected by changes in the hydrogen bonding network due to increasing ionic strength as the titration proceeded. To apply baselines, we utilized Horiba's "Labspec6" software to automatically apply a $7^{th}$ degree polynomial, and within our python processing code we apply a subsequent "morphological baseline" using the "pybaselines" python package. These steps were performed automatically across all spectra. While generating calibration curves, both peak area and maximum peak height of the carbonate and bicarbonate peaks yielded linear responses with respect to concentration indicating nearly constant Gaussian/Lorentzian balance in the line shape. Assuming Beer-Lambert, the spectroscopic data also validated the mole balance; moles of base added equals moles conjugate base which was used to calculate concentrations at each addition step of the titration. Maximum peak height was utilized as our concentration proxy to eliminate any Voigt fitting errors from the process. Notably, we have systematic error in our Voigt fits which indicates the measured peaks are slightly red skewed as seen in Figure SI2. We believe this is due to stark broadening and shifting caused by ion pairing which can give inhomogeneous broadening effects. pH was measured to track the progress of the titrations at each addition step (Fisher Accumet AE150). The range of peak heights and areas for each molecule were normalized between zero and one. The absolute value of the difference between the normalized areas and heights, and thus difference in concentration between the two species, was plotted versus pH. The apparent $pK_a$ should be located at the minimum of that curve, but surprisingly, the apparent $pK_a$ was not the expected value of 10.33, but consistently around 10.1, a substantial deviation. Such a plot is shown in figure SI3. After consideration, we found this deviation to be solely due to the mass action of the equilibrium reactions depending on activity and not concentration. To account for this, the extended Debye-Hückel theory was applied to calculate activity coefficients at each titration step, which reached non-ideal values as low as 0.77 and 0.49 for bicarbonate and carbonate respectively, assuming a hydrated radius of 4.5 Å for both ions. The deviation from ideal behavior is due to the relatively high ionic strength of the nominal concentration of bicarbonate and the increasing ionic strength due to the addition of KOH. This observation then raises

questions regarding local ionic strength and effective local $pK_a$ , which could play pivotal roles in determining proton donors in more complex equilibrium systems, not only in DAC solvents, but also in mixed electrolyte systems near electrocatalytic sites, which in RCC schemes would also act as the DAC solvent. This consideration of local $pK_a$ could play a critical role in determining reaction mechanisms and product distribution[1–3].

Typical spectral mapping spacing was 20 µm by 1000 µm in the X and Y directions, respectively while high resolution maps had spacings of 5 µm by 200 µm respectively. Mapping resolutions approaching low single-digit microns are possible with the optics of the system and the precision of the XY stage, but the spatial resolution scales with the mapping-time squared if the mapped area is held constant. Therefore, we chose mapping resolutions for an optimal balance between speed and resolution. We chose a higher resolution in the X direction because concentration changes in the Y direction are relatively smaller than those in the X direction. For the highest spectral quality, standard "point-by-point" mapping can be performed, during which the stage is moved to a position and then multiple long integration-time captures can be averaged, and the stage moved to the next position until all desired locations are spectrally mapped. Horiba's SWIFT™ capture method performs continuous spectral capturing while simultaneously rastering to facilitate higher throughput with the tradeoff of effectively lower integration times and thus lower signal-to-noise ratio. Using the standard "point-by-point" method the mappings in this work could take up to approximately 10 hours, while the SWIFT method can perform the same mapping in approximately 45 minutes. Given the limited volume available while using a syringe pump, we consider the spectral quality trade-off acceptable.

Given that most DAC solvents follow similar equilibrium cascade reactions, our model framework could be generalized and modified to work with other DAC solvents. It is assumed that liquid flow within the flow cell's measurement chamber is fully developed and laminar and that the velocity profile is known and follows Hagen–Poiseuille flow within the mid-height XY plane where the spectroscopy measurements are taken and that the X velocity component is zero. These flow characteristics were verified via video particle tracking, shown in Figure SI1. It is also assumed that the reactor is at steady state and that the liquid density and diffusivities of chemical species are constant. The transient period to reach steady state was characterized via single-spot transient spectroscopic measurements and those wait times were adhered to with an additional margin for every mapping performed. It is assumed that gas absorption at the gas-liquid interface follows a Henry's law equilibrium-approach similar to two-film-theory, which is to say that the rate of mass transfer of $CO_{2(g)}$ to $CO_{2(aq)}$ at the boundary is determined by difference in concentration of $CO_{2(aq)}$ at the boundary from its equilibrium value determined by Henry's law. In this way, the flux increases or decreases based on the subsequent reactions near the interface and is limited by the diffusivities, kinetics, and the partition coefficient up to the limiting equilibrium case. Using this approach hydroxide ions can be thought to promote the gas absorption process.

The interfacial $CO_2$ mass transfer is captured in the finite difference style boundary condition at the membrane via Henry's law calculation of a ghost point outside the model domain. Thus, the flux is essentially controlled by the removal of $CO_{2(aq)}$ within the domain due to reactions, convection, and diffusion while being capped by reaching equilibrium. This direct treatment is justified by the excessive 200 SCCM flow of $CO_{2(g)}$ within the gas side of the flow cell. The boundary condition equations for the model are shown below in equations 1-7:

$$C_{OH-}\Big|_{top} = C^0_{KOH} \quad (1)$$

$$C_{H+}\Big|_{top} = K_W / C^0_{KOH} \quad (2)$$

$$C_{i \neq OH-,H+}\big|_{top} = 0 \quad (3)$$

$$\frac{\partial C_i}{\partial x}\Big|_{left} = 0 \quad (4)$$

$$\frac{\partial C_{i \neq CO2}}{\partial x}\Big|_{right} = 0 \quad (5)$$

$$\frac{\partial C_{CO2}}{\partial x}\Big|_{right} = \frac{-D_{CO2}\left(h_{CO_2}\, P^{cell}_{CO_2} - C_{CO2}|_{right-2\Delta x}\right)}{2\Delta x} \quad (6)$$

$$\frac{\partial C_i}{\partial y}\Big|_{bottom} = 0 \quad (7)$$

Where $C^0_{KOH}$ is the inlet concentration of KOH, $h_{CO_2}$ is the partition coefficient for $CO_2$ with units of $mol^1\ m^{-3}\ mmH_2O^{-1}$, and $P^{cell}_{CO_2}$ is the partial pressure of $CO_2$ in $mmH_2O$ in the gas channel of the flow cell and is a measured quantity averaging 8300 $mmH_2O$ in our lab with a negligible drop of 30 $mmH_2O$ across the gas channel of the cell. The boundary conditions consist of a constant concentration of KOH and equilibrium $H^+$ at the inlet, zero flux on the left impermeable wall, an inward flux of the dissolved $CO_2$ at the membrane following a finite difference Henry's law equilibrium-approach, and an open boundary at the exit dictated by a zero diffusive flux condition which leaves only convective fluxes at the exit. This system of coupled partial differential equations is exceptionally stiff as the Damköhler and Péclet numbers span orders of magnitude. It was found that the method of lines (MOL) was able to provide acceptable solutions in reasonable times. The MOL is a semi-analytical technique developed to solve boundary value problems in physics[4]. In the 1960's and 1970's, the theory of MOL was developed and shown to reduce many parabolic partial differential equations (PDEs) into a solvable system of ordinary differential equations (ODEs)[5,6]. The concept behind MOL is to slice the system domain into a discrete set of continuous lines leaving only one discrete independent variable over which the finite difference method is used, while an ODE solver can be used in the continuous direction. In our system, we chose to discretize the X direction because the conditions at the top of the reactor are known (constant concentration of KOH) which leads to a system of initial value problems in the Y direction. This system of Y direction ODEs could then be solved stably by the Radau method, so long as the dynamics in the X direction were properly captured by the discretization. The X direction discretization facilitates our treatment of the membrane flux boundary condition via ghost points outside of the reactor solution domain.

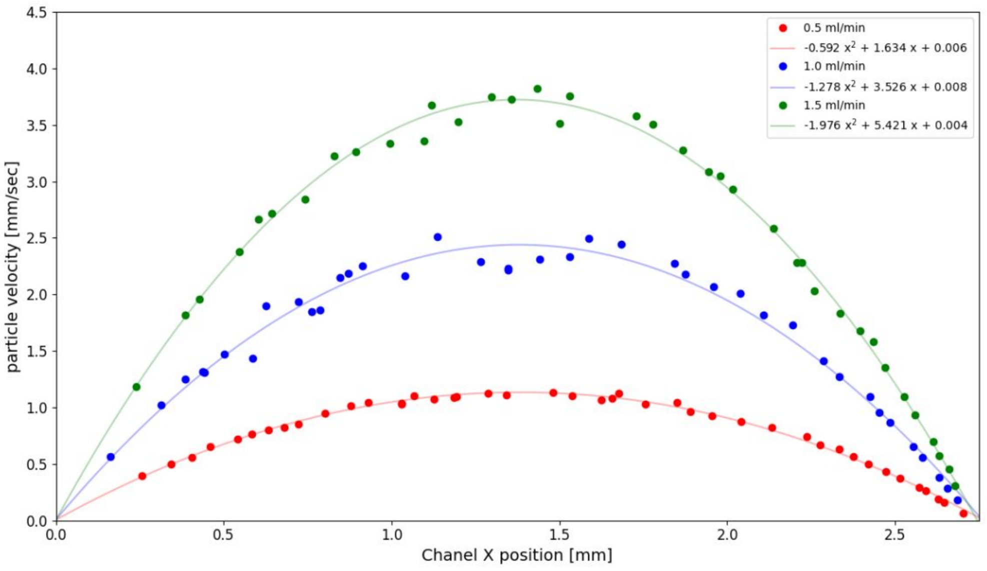


**FIGURE SI1:** Manual particle tracking velocimetry (dots) at 0.5, 1.0 and 1.5 ml/min with good quadratic fits (lines) indicating laminar Hagen-Poiseuille flow. $R^2$ = 0.995, 0.977, and 0.995 respectively.

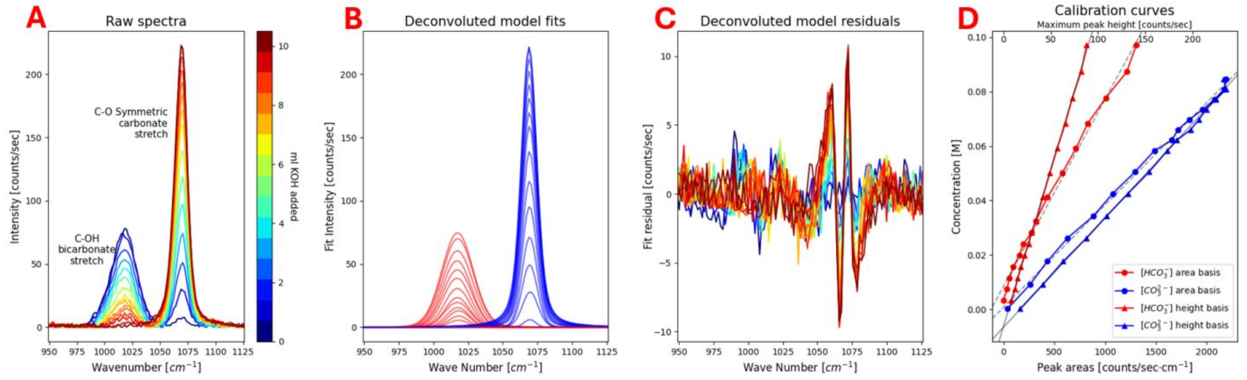


**FIGURE SI2:** A) Raw titration spectra color coded by volume addition of 1M KOH (blue to red) to a nominal solution of 0.1M bicarbonate showing the trade of from bicarbonate to carbonate throughout titration at steps of known concentrations. B) Deconvoluted model spectra for bicarbonate (red) and carbonate (blue) used for area based calibrations. C) full model spectra fit residuals color coded the same as A. Note that the systematic error in the carbonate peak is at worst 5% and is likely due to increasing ionic strength red skewing the peak due to ion paring stark effects. D) Calibration curves based on peak area as well as peak height confirming valid linear relationship between both methods.

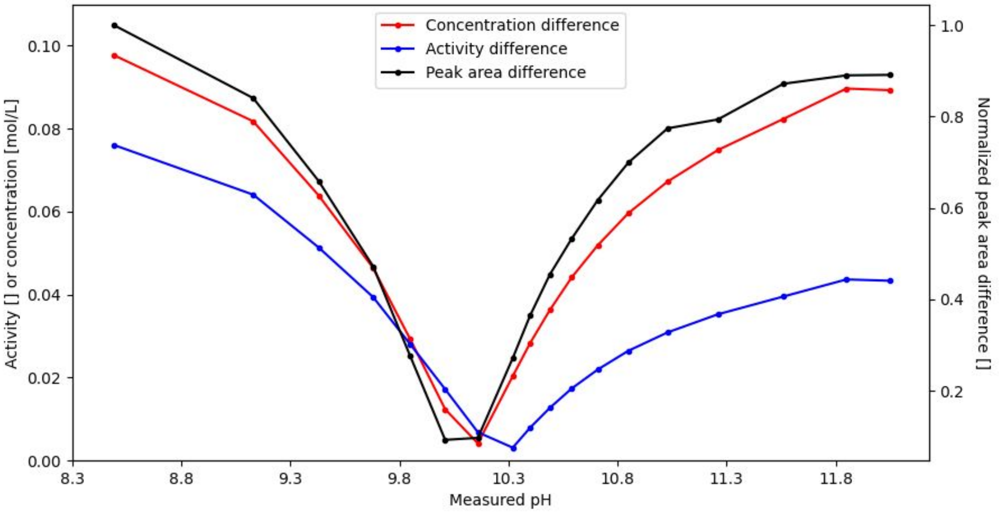


**FIGURE SI3:** calculated values throughout a spectroscopic titration. Red: absolute value of the concentration difference between carbonate and bicarbonate corrected for volume addition and calculated from direct mole balance based on KOH additions. Black: the absolute value of the difference of the normalized range of peak areas for carbonate and bicarbonate which corresponds to an uncalibrated spectroscopic concentration. Blue: absolute value of the difference between activities calculated from mole direct balance (red curve). The minima of each curve should correspond to the pKa; the point where the buffer species are equal. Only the blue curve, activity difference, has a minimum where the pKa of potassium bicarbonate should sit thus highlighting the importance of activity in this system at relatively high concentrations.

$H^+$ "A": $$V_y(x)\frac{\partial C_A}{\partial y} = D_A\left(\frac{\partial^2 C_A}{\partial y^2}+\frac{\partial^2 C_A}{\partial x^2}\right)+k_1^f - k_1^b\gamma_A C_A\gamma_B C_B + k_3^f\gamma_C C_c - k_3^b\gamma_A C_A\gamma_D C_D - k_5^f\gamma_A C_A\gamma_E C_E + k_5^b\gamma_D C_D \quad (8)$$

$OH^-$ "B": $$V_y(x)\frac{\partial C_B}{\partial y} = D_B\left(\frac{\partial^2 C_B}{\partial y^2}+\frac{\partial^2 C_B}{\partial x^2}\right)+k_1^f - k_1^b\gamma_A C_A\gamma_B C_B - k_2^f\gamma_C C_c\gamma_B C_B + k_2^b\gamma_D C_D \quad (9)$$

$CO_2$ "C": $$V_y(x)\frac{\partial C_C}{\partial y} = D_C\left(\frac{\partial^2 C_C}{\partial y^2}+\frac{\partial^2 C_C}{\partial x^2}\right)- k_2^f\gamma_C C_c\gamma_B C_B + k_2^b\gamma_D C_D - k_3^f\gamma_C C_c + k_3^b\gamma_A C_A\gamma_D C_D \quad (10)$$

$HCO_3^-$ "D": $$V_y(x)\frac{\partial C_D}{\partial y} = D_D\left(\frac{\partial^2 C_D}{\partial y^2}+\frac{\partial^2 C_D}{\partial x^2}\right)+k_2^f\gamma_C C_c\gamma_B C_B - k_2^b\gamma_D C_D - k_4^f\gamma_D C_D\gamma_B C_B + k_4^b\gamma_E C_E + k_5^f\gamma_A C_A\gamma_E C_E - k_5^b\gamma_D C_D \quad (11)$$

$CO_3^{2-}$ "E": $$V_y(x)\frac{\partial C_E}{\partial y} = D_E\left(\frac{\partial^2 C_E}{\partial y^2}+\frac{\partial^2 C_E}{\partial x^2}\right)+k_4^f\gamma_D C_D\gamma_B C_B - k_4^b\gamma_E C_E - k_5^f\gamma_A C_A\gamma_E C_E + k_5^b\gamma_D C_D \quad (12)$$

**Equations SI 1-5:** Fully expanded model equations where each chemical species is replaced by a letter name for compactness.

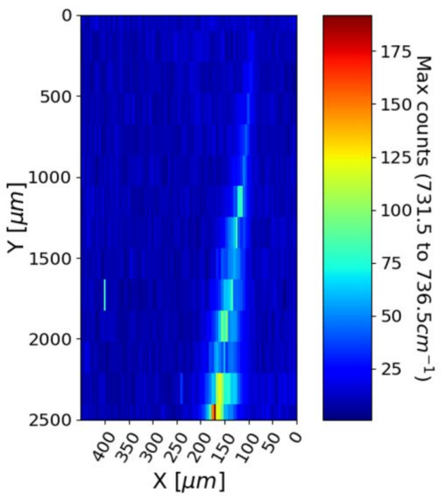


**FIGURE SI4:** Unshifted high resolution map of maximum spectral intensity between 731.5 to 736.5 $cm^{-1}$ corresponding to the PTFE C-F stretch peak. The left side edge of the band in 2D is used to shift-correct each experimental map.

**Table SI1:** model parameters

| Variable | Variable name | Value | Units | Source |
|---|---|---|---|---|
| $D_A$ | Proton diffusivity | $9.31 * 10^{-9}$ | $m^2\ sec^{-1}$ | 7 |
| $D_B$ | Hydroxide diffusivity | $5.27 * 10^{-9}$ | $m^2\ sec^{-1}$ | 7 |
| $D_C$ | Carbon dioxide diffusivity | $1.9 * 10^{-9}$ | $m^2\ sec^{-1}$ | 8 |
| $D_D$ | Bicarbonate diffusivity | $1.18 * 10^{-9}$ | $m^2\ sec^{-1}$ | 7 |
| $D_C$ | Carbonate diffusivity | $0.92 * 10^{-9}$ | $m^2\ sec^{-1}$ | 7 |
| $k_p$ | 2nd order proton transfer reaction rate | $10^8$ | $m^3\ sec^{-1} mol^{-1}$ | 9 |
| $C_W$ | Constant concentration of water | 55,500 | $mol\ \ m^{-3}$ | - |
| $K_W$ | Water autoionization equilibrium constant* | $10^{-8}$ | $(mol\ \ m^{-3})^2$ | - |
| $k_1^f$ | Water autoionization forward rate | $k_1^b * \frac{K_W}{C_W}$ | $sec^{-1}$ | - |
| $k_1^b$ | Water autoionization reverse rate | $k_p$ | $m^3\ sec^{-1} mol^{-1}$ | - |
| $K_2$ | Reaction 2 equilibrium constant | $4.5 * 10^4$ | $mol^{-1}\ \ m^3$ | 10 |
| $k_2^f$ | Reaction 2 forward rate | 9.4 | $m^3\ sec^{-1} mol^{-1}$ | 11 |
| $k_2^b$ | Reaction 2 backwards rate | $\frac{k_2^f}{K_2}$ | $sec^{-1}$ | - |
| $K_3$ | Reaction 3 equilibrium constant** | $7.57 * 10^{-7}$ | $mol\ \ m^{-3}$ | Activity corrected[12] |
| $k_3^f$ | Reaction 3 forward rate** | 0.0388 | $sec^{-1}$ | 13 |
| $k_3^b$ | Reaction 3 backwards rate** | $\frac{k_3^f}{K_3}$ | $m^3\ sec^{-1} mol^{-1}$ | - |
| $K_4$ | Reaction 4 equilibrium constant** | 4.69 | $mol\ \ m^{-3}$ | 10 |

| | | | | |
|---|---|---|---|---|
| $k_4^f$ | Reaction 4 forward rate** | $k_p$ | $m^3\ sec^{-1} mol^{-1}$ | - |
| $k_4^b$ | Reaction 4 backwards rate** | $\frac{k_4^f}{K_4}$ | $sec^{-1}$ | - |
| $K_5$ | Reaction 5 equilibrium constant | $2.13 * 10^7$ | $mol\ m^{-3}$ | [10] |
| $k_5^f$ | Reaction 5 forward rate | $k_p$ | $m^3\ sec^{-1} mol^{-1}$ | - |
| $k_5^b$ | Reaction 5 backwards rate | $\frac{k_5^f}{K_5}$ | $sec^{-1}$ | - |
| $h_{CO2}$ | $CO_2$ partition coefficient | $0.0033$ | $mol\ m^{-3}\ mm_{H_2O}^{-1}$ | [14] |
| $P_{CO2}^{cell}$ | $CO_2$ pressure in gas side of reactor | $8500$ | $mm_{H_2O}$ | measured |

* treating $K_W$ as the ion concentration product in units of $mol\, m^{-3}$ for $H^+$ and $OH^-$. All equilibrium constants are constructed using concentrations in $mol\, m^{-3}$ and not unitless activities.

** Reactions other than reaction 1 treat water implicitly.

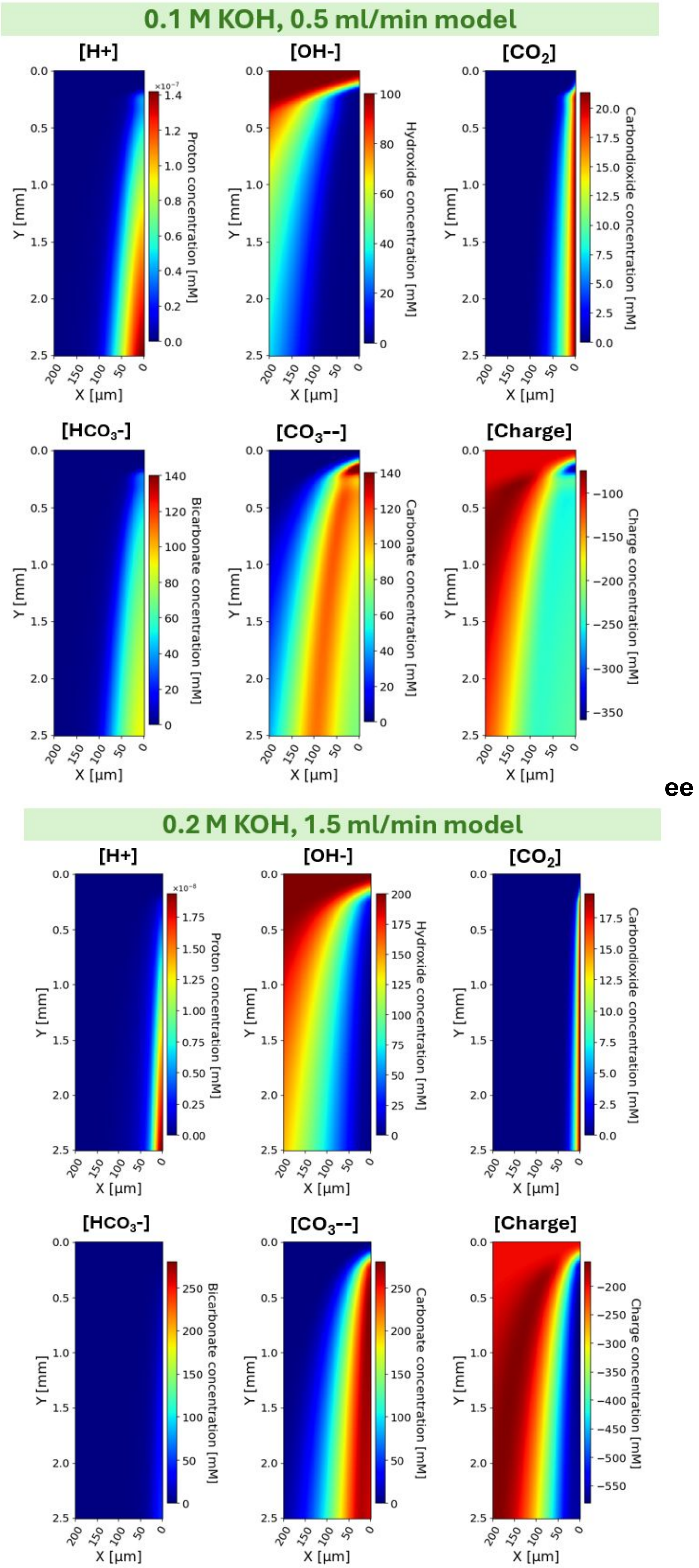


**FIGURE SI5:** All plots from one model run at 0.5 ml/min flow rate and 0.1M KOH and 0.2M KOH at 1.5 ml/min. Note the charge plot and the deviation from electroneutrality.

(1) Zhang, Z.; Melo, L.; Jansonius, R. P.; Habibzadeh, F.; Grant, E. R.; Berlinguette, C. P. pH Matters When Reducing CO2 in an Electrochemical Flow Cell. *ACS Energy Lett.* **2020**, *5* (10), 3101–3107. https://doi.org/10.1021/acsenergylett.0c01606.

(2) Leverick, G.; Bernhardt, E. M.; Ismail, A. I.; Law, J. H.; Arifutzzaman, A.; Aroua, M. K.; Gallant, B. M. Uncovering the Active Species in Amine-Mediated CO2 Reduction to CO on Ag. *ACS Catal.* **2023**, *13* (18), 12322–12337. https://doi.org/10.1021/acscatal.3c02500.

(3) Marquez, R. A.; Bender, J. T.; da Cunha, S. C.; Aleman, A. M.; Sahu, A.; Ganesan, V.; Milliron, D. J.; Resasco, J.; Jaramillo, T. F.; Mullins, C. B. Tracking Local pH Dynamics during Water Electrolysis via In-Line Continuous Flow Raman Spectroscopy. *ACS Energy Lett.* **2025**, *10* (4), 2075–2083. https://doi.org/10.1021/acsenergylett.5c00582.

(4) Sadiku, M. N. O.; Obiozor, C. N. A Simple Introduction to the Method of Lines. *International Journal of Electrical Engineering & Education* **2000**, *37* (3), 282–296. https://doi.org/10.7227/IJEEE.37.3.8.

(5) Zafarullah, A. Application of the Method of Lines to Parabolic Partial Differential Equations With Error Estimates. *J. ACM* **1970**, *17* (2), 294–302. https://doi.org/10.1145/321574.321583.

(6) Sarmin, E. N. Application of the Method of Straight Lines to the Solution of Boundary Value Problems for Certain Non-Selfconjugate Two-Dimensional Second Order Elliptic Equations. *USSR Computational Mathematics and Mathematical Physics* **1965**, *5* (5), 240–246. https://doi.org/10.1016/0041-5553(65)90020-0.

(7) PHREEQC Version 3 | U.S. Geological Survey. https://www.usgs.gov/software/phreeqc-version-3 (accessed 2025-11-05).

(8) Cadogan, S. P.; Maitland, G. C.; Trusler, J. P. M. Diffusion Coefficients of CO2 and N2 in Water at Temperatures between 298.15 K and 423.15 K at Pressures up to 45 MPa. *J. Chem. Eng. Data* **2014**, *59* (2), 519–525. https://doi.org/10.1021/je401008s.

(9) Eigen, M. A. Immeasurably Fast Reactions.

(10) CRC Handbook of Chemistry and Physics, 84th Edition Edited by David R. Lide (National Institute of Standards and Technology). CRC Press LLC: Boca Raton. 2003. 2616 Pp. $139.95. ISBN 0-8493-0484-9. *J. Am. Chem. Soc.* **2004**, *126* (5), 1586–1586. https://doi.org/10.1021/ja0336372.

(11) Pinsent, B. R. W.; Pearson, L.; Roughton, F. J. W. The Kinetics of Combination of Carbon Dioxide with Hydroxide Ions. *Trans. Faraday Soc.* **1956**, *52*, 1512. https://doi.org/10.1039/tf9565201512.

(12) Zeebe, R.; Wolf-Gladrow, D. CO2 in Seawater: Equilibrium, Kinetics, Isotopes; 2001.

(13) Johnson, K. S. Carbon Dioxide Hydration and Dehydration Kinetics in Seawater. *Limnology and Oceanography* **1982**, *27* (5), 849–855. https://doi.org/10.4319/lo.1982.27.5.0849.

(14) Carroll, J. J.; Slupsky, J. D.; Mather, A. E. The Solubility of Carbon Dioxide in Water at Low Pressure. *J. Phys. Chem. Ref. Data* **1991**, *20* (6), 1201–1209. https://doi.org/10.1063/1.555900.
